# Looks can be Deceiving: A Challenging Case of Anti-Neutrophil Cytoplasmic Autoantibody Associated Vasculitis

**DOI:** 10.7759/cureus.15906

**Published:** 2021-06-24

**Authors:** Bashar Ramadan, Jocelyn Taylor, Moeed Ahmed, Eric K Magliulo, Khalid Bashir

**Affiliations:** 1 Internal Medicine, Creighton University School of Medicine, Omaha, USA; 2 Nephrology, Creighton University School of Medicine, Omaha, USA

**Keywords:** hydralazine, anca associated vasculitis, : acute kidney injury

## Abstract

[Anti-neutrophil cytoplasmic autoantibody (ANCA)-associated vasculitis] (AAV) is an autoimmune disease characterized by systemic vascular inflammation. We present a case of a 76-year-old man who presented with shortness of breath, fatigue, and weakness. He was eventually diagnosed with hydralazine-induced ANCA-associated renal limited glomerulonephritis. The presentation of this case was unique for a few reasons; the patient showed an initial improvement in kidney function, was non-oliguric, and had no systemic signs of vasculitis. This led to the patient being discharged prematurely with the diagnosis of acute tubular necrosis. We discuss educational features of this case and warn future clinicians about the possibility of waxing and waning renal function in these patients, as well as the importance of having a higher index of suspicion for glomerulonephritis in patients who take hydralazine.

## Introduction

[Anti-neutrophil cytoplasmic autoantibody (ANCA)-associated vasculitis] (AAV) is an autoimmune disease characterized by systemic vascular inflammation. The spectrum of AAV ranges from the renal limited variant to multi-organ system disease involvement [1]. All forms of AAV have the potential to cause kidney failure secondary to rapidly progressive glomerulonephritis (RPGN). About 20%-40% of AAV cases with kidney involvement develop end-stage renal failure [2]. These patients typically show a persistent and progressive decline of renal function manifested by worsening serum creatinine over a short span of days to months with accompanied oliguria [3].

Hydralazine is a commonly prescribed antihypertensive agent. It is known to be implicated in drug-induced lupus (DIL). AAV is less commonly associated with hydralazine, however, has been shown to be more deadly than DIL. Hydralazine-induced vasculitis has an incidence of 5.4% in patients on 100 mg/day for greater than three years duration. Hydralazine is the most widely associated medication with drug-induced AAV [4-5].

We present a unique case of renal limited glomerulonephritis likely caused by hydralazine-induced AAV. This case demonstrates that some expected clinical features may not always be present, and this can lead to a possible delay in diagnosis as well as a potential increase in morbidity for these patients.

## Case presentation

This is a case of a 76-year-old Caucasian man with a known past medical history of chronic kidney disease stage IIIb and hypertension, who had been taking hydralazine, losartan, and hydrochlorothiazide for several years. He presented with complaints of shortness of breath, fatigue, weakness, and decreased oral intake for two weeks prior to admission. He denied any symptoms of arthralgia, myalgia, cough, and headache. Physical examination was relatively unremarkable; the patient did not have any signs of hypervolemia, the integumentary exam was normal with no visible rash, and lungs were clear to auscultation. His chest X-ray was negative for pulmonary edema or other acute processes. Serum troponin and D-dimer levels were within the normal limit. The patient was non-oliguric and was making around two liters of urine per day.

Other laboratory findings were consistent with an acute or chronic kidney injury. His serum creatinine was 6.5 mg/dL, up from his baseline of 1.6 mg/dL. Urinalysis was positive for microscopic hematuria with 10-20 red blood cells (RBC) per high power field (HPF) as well as an elevated protein/creatinine of 0.9. A serologic workup including antinuclear antibody (ANA), C3, C4, anti-glomerular basement membrane antibody (anti-GBM Ab), hepatitis B surface antigen (HBsAg), antibody to hepatitis B surface antigen (HBsAb), and hepatitis B core antibody (HBcAb) was unremarkable. However, the p-ANCA titer was high at 1:160. Serum and urine immunofixation electrophoresis were negative for monoclonal protein. Renal ultrasound was unremarkable. He was treated with IV fluids, and his aforementioned antihypertensives, which included hydralazine, were discontinued. Interestingly, he continued to have good urine output as well as improvement in his symptoms and his serum creatinine dropped to 4.8 mg/dL. Given the improvement of his renal function and clinical symptoms, he was discharged to close follow-up with the nephrology clinic and his hydralazine was resumed.

**Figure 1:**
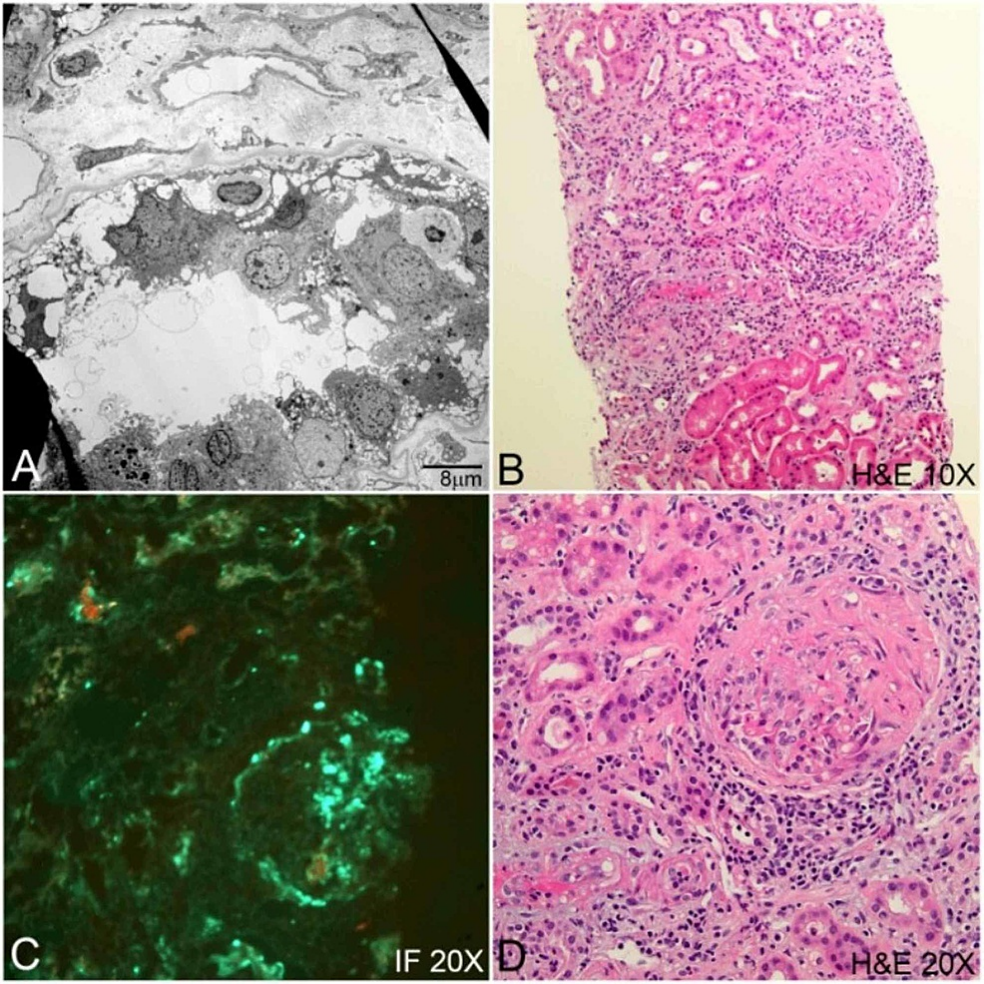
ANCA vasculitis. A, podocyte foot process damage and fall off seen on electron microscopy. B, crescent in glomeruli as well as renal tubular injury and interstitial inflammation seen on light microscopy. C, fibrinogen deposits within the crescents seen on immunofluorescence. D, crescentic glomerulonephritis seen on light microscopy

Follow-up blood work in two weeks showed worsening serum creatinine to 6.8 mg/dL. He had persistent microscopic hematuria. Repeat serologic workup revealed a p-ANCA titer of 1:320 and an elevated myeloperoxidase immunoglobulin G (MPO IgG) of 78 U/mL. The patient was readmitted to the hospital for further workup. A renal biopsy was obtained, which revealed pauci-immune glomerulonephritis, including crescent formation, segmental necrotizing inflammation on light microscopy, and absence of immune deposits on immunofluorescence (Figure 1). Steroids and rituximab were initiated as treatment. Hydralazine, which was re-initiated at the time of discharge, was now discontinued on the suspicion of being an etiologic agent of AAV. However, there is a possibility that this was ANCA-associated vasculitis unrelated to hydralazine. Due to worsening kidney function, the patient was started on hemodialysis. He remained dialysis-dependent with no significant renal recovery.

## Discussion

The early diagnosis of AAV is important in minimizing dialysis dependence and reducing mortality. Treatment modalities such as rituximab, cyclophosphamide, and steroids have been shown to be effective in these patients [6-9]. However, these treatments are not entirely benign and come at a cost of prolonged immunosuppression. For this reason, the diagnostic modality of choice is a confirmatory kidney biopsy which typically shows pauci-immune crescentic glomerulonephritis. This procedure is relatively safe compared to biopsies of other organs with only a 3.2% chance of major complications [10-11]. However, before proceeding with a kidney biopsy an exhaustive workup supporting a diagnosis of glomerulonephritis has to be established.

What made this case particularly educational is the unusual clinical presentation that led to a delay in renal biopsy. One unusual feature was the initial improvement of kidney function with supportive measures. The fact that this patient’s serum creatinine levels significantly improved with IV fluid alone is not typical for rapidly progressive glomerulonephritis (RPGN). Normally RPGN would show a persistently worsening kidney function and decreased urine output [3]. This patient did not have oliguria and was consistently producing adequate amounts of urine. Volume overload can cause a dilutional effect on serum creatinine; however, this patient’s creatinine improved more than what was expected from dilution alone. He did not show signs of volume overload and had improvement in urine output. Another unique feature of this case was the lack of systemic signs. AAV normally has systemic signs of vasculitis that can lead towards a diagnosis of glomerulonephritis, however, it is reported that AAV can also rarely present as renal limited glomerulonephritis [12]. The initial improvement in renal function and the lack of features suggestive of vasculitis supported a diagnosis of acute tubular necrosis and this led to the discharge of the patient and a two-week delay in obtaining the kidney biopsy. The persistence of microscopic hematuria, the detection of p-ANCA with increased titers, and the re-worsening of serum creatinine increased the suspicion of RPGN enough to pursue a biopsy which secured the diagnosis of renal limited glomerulonephritis.

This patient was taking hydralazine for his hypertension which we think was the likely etiologic agent for AAV. Given the non-specific clinical presentation and rarity of cases, establishing a diagnosis of hydralazine-induced AAV can pose a significant challenge. Current evidence from the literature shows no clearly defined relationship between the initiation and duration of exposure to hydralazine and the onset of glomerulonephritis. In fact, patients can present after several years of exposure to hydralazine [13]. One possible explanation for the patient’s initial improvement in serum creatinine was the discontinuation of hydralazine in his first admission and the re-initiation at discharge. High titers of anti-MPO antibodies are associated with drug-induced glomerulonephritis and serve as an essential diagnostic clue in an often puzzling clinical picture [14]. The discontinuation of the offending drug earlier on is likely to be important in allowing for renal recovery and so a higher clinical suspicion for glomerulonephritis should be maintained in these patients.

## Conclusions

In conclusion, this case demonstrates pitfalls clinicians should be aware of when it comes to diagnosing AAV. Waxing and waning kidney function may occur in AAV, therefore, initial improvement of kidney function does not preclude a diagnosis of glomerulonephritis even in patients with good urine output. Renal limited glomerulonephritis is a possibility with AAV and so the lack of systemic signs should not be surprising, and lastly, patients using hydralazine should have a higher index of suspicion for AAV especially when presenting with unexplained microscopic hematuria and proteinuria.
